# Empirical validation of ELM trained neural networks for financial modelling

**DOI:** 10.1007/s00521-022-07792-3

**Published:** 2022-10-01

**Authors:** Volodymyr Novykov, Christopher Bilson, Adrian Gepp, Geoff Harris, Bruce James Vanstone

**Affiliations:** 1 Bond Business School, Bond University, Gold Coast, QLD Australia; 2 Bangor Business School, Bangor University, Wales, UK

**Keywords:** Extreme Learning Machine, Stock price prediction, Long Short-Term Memory, Recurrent Neural Network

## Abstract

The purpose of this work is to compare predictive performance of neural networks trained using the relatively novel technique of training single hidden layer feedforward neural networks (SFNN), called Extreme Learning Machine (ELM), with commonly used backpropagation-trained recurrent neural networks (RNN) as applied to the task of financial market prediction. Evaluated on a set of large capitalisation stocks on the Australian market, specifically the components of the ASX20, ELM-trained SFNNs showed superior performance over RNNs for individual stock price prediction. While this conclusion of efficacy holds generally, long short-term memory (LSTM) RNNs were found to outperform for a small subset of stocks. Subsequent analysis identified several areas of performance deviations which we highlight as potentially fruitful areas for further research and performance improvement.

## Introduction

The prediction of stock price movements is of particular interest to traders in financial markets, in addition to a wide range of finance applications not limited to the market timing strategies of funds managers and the selective hedging practices of corporations. Paradoxically the most successful theories of this field assume that stock prices undergo random walks when markets are in equilibrium and include work pioneered by the seminal publications of Black, Scholes [2, 3], and Merton [16] in continuous time and the Binomial Options Pricing model of Cox, Ross, and Rubinstein [7] in discrete time. Nonetheless, this remains a developing area, with frequent deviations from the random walk assumption observed (such as those identified in Jacobs [14]) and with recent advances in data provision and computational techniques many of these approaches have found application in this area of research.

One such application has been machine learning techniques (Strader (2020)). This field has seen the widespread application of Neural Network models, which are explicitly nonlinear in their mathematical representations (see, for example, Chollet [6] with such specifications as Long Short-Term Memory (LSTM) models being particularly suited to financial time-series analysis. Nevertheless, much of the published work is accomplished using deep neural networks, with relatively few studies using shallow neural networks notably across regionally separated markets. Strader [23] in particular, highlights these areas as requiring additional empirical research. The Extreme Learning Machine (ELM) of Huang et al. [13] is one such alternative training technique for shallow neural networks that has recently seen promising empirical results across various practical applications. These applications have been in diverse areas of research, ranging from image quality assessment Suresh, Venkatesh Babu, and Kim [25], improving wireless sensor network localization Phoemphon, So-In, and Niyato [21] to predicting landslides (KV, Pillai, and Peethambaran [17]). A natural extension of these applications then is in the area of financial research.^1^

Tang et al. [27] present a case for using a Random Vector Functional Link (RVFL) network, a special case of single hidden layer feedforward neural networks, together with a Variational Mode Decomposition for the day-ahead crude oil price prediction and compare the results with other neural networks. They find the RVFL network outperforms simpler ELM-based models (as it distinctively includes a direct input–output link, unlike an ELM), albeit taking longer to train. Bisoi et al. [4, 5] support these findings on the same dataset utilising similar network choices. Bisoi et al. [4, 5] explore the same concept of Variational Mode Decomposition yet applying it in the study of next-day ahead stock price prediction using ELM-based models. They analyse a sample of daily prices from regional markets and find promising results for the S&P BSE 500, Hang Seng and FTSE 100 Indices. Mohanty, Parida, and Khuntia [19] show an improvement on a plain ELM-based model performance for stock price prediction by morphing it into a kernel ELM (KELM) combined with an auto encoder (AE). Mohanty et al. [19] test this augmented model (KELM-AE) on several bank stock indices using a normalised OHLC (open-high-low-close) “candlestick” dataset as input variables with the next-day ahead prediction. Göçken et al. [10] compare the effectiveness of using ELM-trained SFNN to predict stock prices on the Borsa Istanbul (BIST 100) stock exchange, albeit focusing on a relatively narrow subset of 3 stocks. Results are compared to other popular models used for the task such as DNN, Jordan RNN, GLM, RT, and Gaussian Process Regression. The conclusion drawn on the best performing model architectures and variables is used for prediction and comparison in this study. An empirical study conducted by Zhang [30] involved an attempt to apply an ELM-trained SFNN to predict price movements of a stock on the Hong Kong stock exchange. Zhang’s results (2021) tantalizingly provide some empirical support to the work of Gocken et al. [10].

This paper utilises emerging findings from Zhang [30] within the framework of Gocken [10] and designs, validates and evaluates a series of SFNN models, trained using the ELM methodology, on the 20 largest stocks on the Australian equity market, also known as constituents of the ASX20. The ELM-trained model results are shown to indeed fulfil the promise of fast learning times (as compared to training the same models using classic backpropagation algorithm) with a comparably high, and often superior, level of accuracy. In general, improving prediction accuracy in this task, even if by a slight margin, can bring about material benefits to the interested stakeholders. This is the driving motivation behind this study; it shows that less computationally intensive model training techniques can deliver potentially higher economic benefits to capital market participants. With varying degree of computational power availability among market participants, designing more efficient techniques, such as those based on ELM training methodology, has direct industry applications. Additionally, performance findings may be used to academically interpret the operating mechanism of neural networks, thus advancing this strand of research.

We draw particular distinction between existing studies in the underlying dataset used as individual stocks are likely to possess different movement characteristics driven by inherent risks and influential factors, as compared to, for example, combinatory stock indices or foreign exchange rates. To the best of our knowledge, there has been no study comparing stock price prediction performance of the most commonly known and successfully used LSTM to the relatively novel ELM training methodology on a broad set of large and frequently traded stocks.

Another differentiating element between these relevant studies is the extent of initial data reconstruction applied to the raw financial price series. Given the well-studied noisiness and dynamics in the financial price series, such structured methods as Empirical or Variation Mode Decomposition Das et al. [8], Bisoi et al. [4, 5], and Discrete Wavelet Transform Wu et al. [28] have previously been applied to the raw stock price series. The rest of the studies rely on the logic of the Takens’ theorem [26], either explicitly or implicitly, by constructing technical features and statistical metrics from the raw price series to be used as the model input Khuwaja et al. [15] provide the most comprehensive detail on the application of this methodology, Das et al. [9], Mohanty et al. [19], Panda et al. [20] appear to implicitly follow this path). We apply the latter approach of explicitly applying the spirit of the Takens’ theorem by constructing technical features from the raw price series, intentionally very similar to the ones used in Zhang [30], albeit with slight modification and an addition.

This paper is organised as follows. Section 2 details the training and testing methodology and data used in this study. Subsequent Results and Discussion section evaluates model performance for the two mentioned training methodologies on the holdout test dataset. Finally, Conclusion and Future Research section concludes the paper and identifies promising future research opportunities.

## Methodology

This section first discusses the models construction and training methodology, followed by the data description and preparation.

### Models and training methodology

#### Extreme learning machine (ELM)

Extreme Learning Machine, as introduced by Huang et al. [13], modifies the training methodology for single hidden layer feedforward neural networks by converting it to an analytical solution. It has been shown ELMs (i.e., ELM-trained SFNNs) produce similar, if not better results for the typical NN-allocated tasks, albeit at much faster training speed and avoiding local optima convergence challenges.

In an ELM, the vectors of input weights and hidden node biases are first randomly assigned and used to calculate the hidden layer output matrix, $H$, using a specified transfer function. The Moore–Penrose generalised inverse of this matrix $H$, denoted as ${\text{H}}^{\dag }$ as adapted from Huang et al. [13], is then used to analytically determine the vector of output weights $\gamma$ that would best fit with the data output matrix $T$.

Mathematical representation of a single hidden layer feedforward neural network (SFNN) trained with Extreme Learning Machine (ELM) training methodology is as follows. A model with $N$ distinct samples and $j$ randomly assigned hidden neurons $({X}_{j},{t}_{j})$ where ${X}_{j}={\left[{x}_{j1},{x}_{j2},\dots ,{x}_{jn}\right]}^{T}$ as an input and $T={\left[{t}_{j1},{t}_{j2},\dots ,{t}_{jn}\right]}^{T}\in {R}^{n}$ as targets vector can be represented by the following equation:

1 $$\mathop \sum \limits_{j = 1}^{L} \gamma_{j} h\left( {W_{j} .X_{n} + b_{j} } \right) = o_{n} , \;for\; n = 1,...,N$$

where ${\gamma }_{j}$ and ${W}_{j}={\left[{w}_{j1},{w}_{j2},\dots ,{w}_{jn}\right]}^{T}$ are the output and input weights, respectively, ${b}_{j}$ is the randomly assigned bias of $jth$ hidden neuron and $h(.)$ is the nonlinear activation function. The goal of the ELM training methodology is to reduce the error between the predicted and the target (actual) values, such that ${\sum }_{n=1}^{N}\Vert {o}_{n}-{t}_{n}\Vert =0$ and ${\sum }_{j=1}^{L}{\gamma }_{j}h\left({W}_{j}.{X}_{n}+{b}_{j}\right)={t}_{n}, for n=1,\dots ,N.$

In short, the ELM training methodology comprises the following steps:

1. Randomly assign hidden layer weights ${W}_{j}$ and bias ${b}_{j}$ values.
2. Calculate the hidden layer output matrix *H*:
  2 $$H = \left[ {\begin{array}{*{20}c} {h\left( {x_{1} } \right)} \\ \vdots \\ {h(x_{N} )} \\ \end{array} } \right] = \left[ {\begin{array}{*{20}c} {h_{1} \left( {W_{1} x_{1} + b_{1} } \right)} & \cdots & {h_{L} \left( {W_{L} x_{1} + b_{L} } \right)} \\ \vdots & \ddots & \vdots \\ {h_{1} \left( {W_{1} x_{N} + b_{1} } \right)} & \cdots & {h_{L} \left( {W_{L} x_{N} + b_{L} } \right)} \\ \end{array} } \right]$$
  where ${h}_{j}(x)$ stands for the nonlinear transfer function of the *j*-th hidden neuron, and *L* stands for the number of hidden neurons chosen in the SFNN.
  Each column of the *H* output matrix represents the *j*-th hidden neuron output vector with regards to the vector of inputs ${x}_{1}, {x}_{2}, \dots , {x}_{N}$.
3. Calculate the vector of output weights ${\gamma }_{j}$:
  3 $$\gamma = {\text{H}}^{\dag} T$$

where $\gamma ={\left[{\gamma }_{1},{\gamma }_{2},\dots , {\gamma }_{L}\right]}^{T}$ is the vector of output weights, $T={\left[{t}_{1},{t}_{2},\dots , {t}_{N}\right]}^{T}$ represents the output matrix, and ${\text{H}}^{\dag}$ is the Moore–Penrose generalised inverse of the hidden layer output matrix *H*.

Effectively, the objective function of the SFNN trained using ELM methodology is the minimisation of the cost function as follows:

4 $$H\left( {\hat{W}_{j} ,\hat{b}_{j} } \right)\hat{\gamma } - T = \mathop {\min }\limits_{W, b, \gamma } \left\| {H\left( {W_{j} ,b_{j} } \right)\gamma - T} \right\|$$

where $j=1,\dots ,L$. The minimisation of the cost function is based on the sum of squared errors calculation represented by $\epsilon$ and is defined in Eq. (5)

5 $$\varepsilon = \mathop \sum \limits_{n = 1}^{N} \left( {\mathop \sum \limits_{j = 1}^{L} \gamma_{j} h\left( {W_{j} .X_{n} + b_{j} } \right) - t_{n} } \right)^{2}$$

**Fig. 1:**
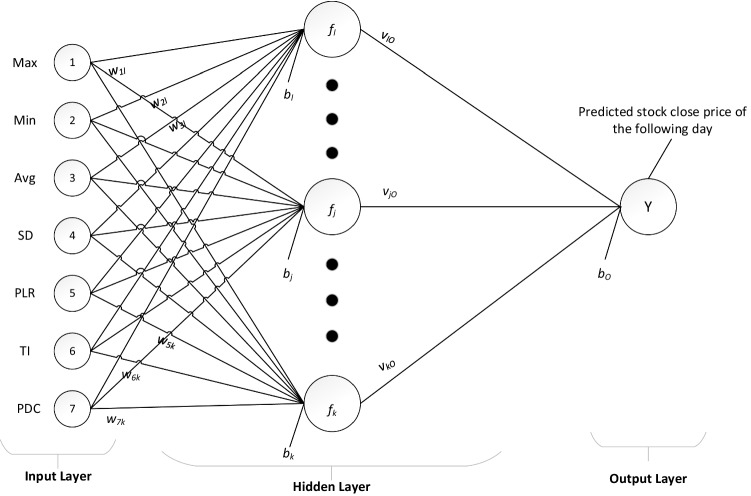
Structure of the ELM

Figure 1 depicts a high level overview of the ELM-trained neural network structure, with the specific inputs and outputs chosen for this study. For full details and proof of theorems underpinning the ELM training methodology please see Huang et al. [13].

“The design of an ANN [*Artificial Neural Network (ANN)*] is more of an art than a science” Zhang, Patuwo and Hu [31], and, in the case of the ELM-trained SFNN, it is mainly the number of hidden nodes in the single hidden layer that needs to be chosen. Some works in this area focus on developing theoretical bounds on the minimum and maximum number of hidden nodes required (for example, LeCun et al. [18] while others develop complex algorithms for supporting this decision using the dataset at hand Xu and Chen [29]. Yet another category focuses on providing rule-of-thumb advice for determining optimal number of hidden nodes based on the number of inputs and outputs in the model, or merely on the number of training samples. Major risk here is discounting some of the other key attributes of the dataset used (e.g., signal-to-noise ratio or complexity of the function to be learnt) that may materially impact on this decision.

Thus, we use cross-validation to determine the optimal number of hidden nodes in the network. Given we are dealing with the time-series data, we split the training set (~ 7.5 years out of the total 10 years of data obtained) into training (the first 5 years) and validation (the remaining ~ 2.5 years) subsets. We train ELM neural networks starting with very small number of hidden nodes (2) progressing incrementally (initially, with increments of 1) to larger network sizes (> 300 hidden nodes in the largest network) for each individual stock. The best topology is then chosen based on the mean squared error results from the validation dataset for each stock across networks trained. Before the best chosen model is used for the final test on the holdout data (the last 2.5 years of data available), training and validation datasets are combined, and this final model is trained on the full training dataset.

#### Recurrent neural network (LSTM)

The Long Short-Term Memory (LSTM) algorithm was developed by Hochreiter and Schmidhuber [11] to address the *vanishing gradient problem*, a persistent effect observed in simpler (i.e., non-recurrent) feedforward neural networks as their depth increases (theoretical explanation behind this effect is discussed extensively by Bengio et al. [1].

At its core, the LSTM algorithm allows the network to “carry” information across many timesteps (hence the name) to be later “reinjected” back into the network when needed. This is particularly useful for tackling tasks where time-series are studied and locally learnt features at some previous point can then be “remembered” by the network and used later when a similar pattern arises. Hu et al. [12] conduct a survey of literature studying deep learning models used for stock price prediction and conclude hybrid LSTM-based models are most widely researched.

We train LSTM neural network models using raw stock daily close price data with identical lookback period of 5 days. Decision to use the raw daily stock price data is based on understanding of the feature extraction mechanism of an LSTM Recurrent Neural Network (RNN). An LSTM network is designed to be able to remember information and features learned several timesteps before the current unit processing which is what we are attempting to accomplish with feature extraction for the ELM-trained SFNNs. 5 day lookback period is chosen to provide direct comparison between ELM-trained SFNN and LSTM neural networks. A three-layer stacked LSTM structure with 50 hidden nodes in each layer is used as a commonly used architecture of this type of a neural network in finance research (see, e.g., Sirignano and Cont [22].

**Fig. 2:**
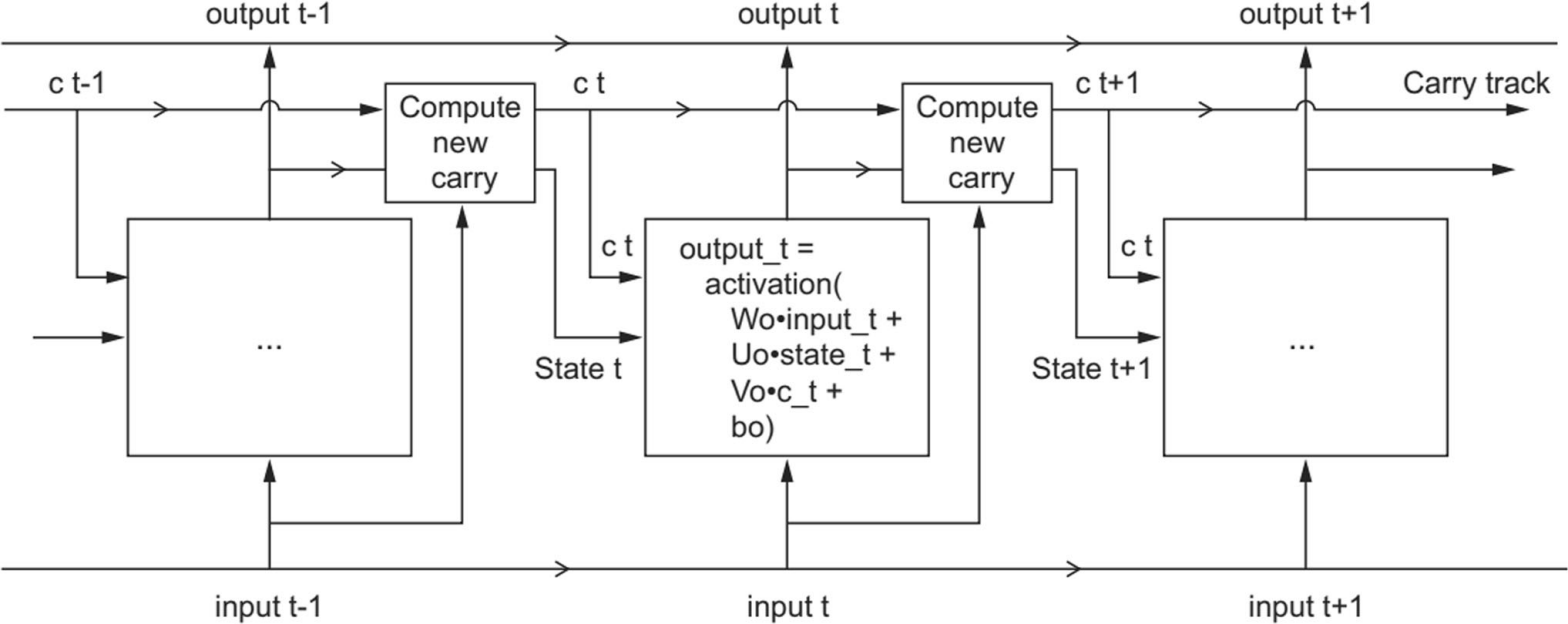
Anatomy of an LSTM *Source:* Chollet [6], p. 204

Figure 2 provides a schematic overview of the training process behind an LSTM-based neural network.

### Data

Individual stocks in the S&P/ASX20 index are chosen for empirical evaluation of the models. This is consistent with existing literature in the field (as discussed in detail in Sect. 1) while also capturing broad coverage of industries on the Australian share market. Daily candlestick chart price data (Open-High-Low-Close, without Volume) is obtained using *yahoo-finance* module in Python (Table 1).

The period under analysis is chosen between the two most significant events of the past 20 years that had major impact on financial markets—Global Financial Crisis and the Covid-19. Thus, 1 January 2010 to 31 December 2019 forms the analysis period, and the price data is split into training, validation, and testing datasets to create an objective evaluation protocol.

Maintaining the sequential logic of the time-series, the first 5 years of the period (1 Jan 2010–31 Dec 2014) form the training dataset, the following 2.5 years are used as the validation dataset (1 Jan 2015–30 Jun 2017), with the remainder 2.5 years (1 Jul 2017–31 Dec 2019) being used as the holdout test dataset for the models.

History of the index constituents for the period under study is obtained and stocks that have been present in the index for more than a year are chosen. Table 2 contains descriptive list of the dataset obtained, and the final set of individual stocks chosen for model testing.

**Table 1:** Study dataset—an overview

Dataset	Full data period	Dataset date split		
		Training data	Validation data	Test/Holdout data
S&P/ASX 20 individual stocks	10 years	1 January 2010–31 December 2014	1 January 2015–30 June 2017	1 July 2017–31 December 2019

**Table 2:** Stock price time-series used in the study

S&P/ASX 20 historical constituent	Company name	Tenure as an ASX 20 member (during study period)	Listed on ASX during entire study period	Included in the study
AMP AU	AMP Limited	Jan-10–May-18	Yes	Yes
ANZ AU	Australia and New Zealand Banking Group Limited	Jan-10–Dec-19	Yes	Yes
BHP AU	BHP Group Ltd	Jan-10–Dec-19	Yes	Yes
BXB AU	Brambles Limited	Jan-10–Dec-19	Yes	Yes
CBA AU	Commonwealth Bank of Australia	Jan-10–Dec-19	Yes	Yes
CSL AU	CSL Limited	Jan-10–Dec-19	Yes	Yes
MQG AU	Macquarie Group Ltd	Jan-10–Dec-19	Yes	Yes
NAB AU	National Australia Bank Ltd	Jan-10–Dec-19	Yes	Yes
NCM AU	Newcrest Mining Ltd	Jan-10–Feb-14, Dec-19	Yes	Yes
ORG AU	Origin Energy Ltd	Jan-10–Feb-16, Jun-18–Nov-18	Yes	Yes
QBE AU	QBE Insurance Group Ltd	Jan-10–Feb-18	Yes	Yes
RIO AU	Rio Tinto Limited	Jan-10–Dec-19	Yes	Yes
SUN AU	Suncorp Group Ltd	Jan-10–Dec-19	Yes	Yes
TLS AU	Telstra Corporation Ltd	Jan-10–Dec-19	Yes	Yes
WBC AU	Westpac Banking Corp	Jan-10–Dec-19	Yes	Yes
WES AU	Wesfarmers Ltd	Jan-10–Dec-19	Yes	Yes
WOW AU	Woolworths Group Ltd	Jan-10–Dec-19	Yes	Yes
WPL AU	Woodside Petroleum Limited	Jan-10–Dec-19	Yes	Yes
STO AU	Santos Ltd	Dec-11–Aug-14	Yes	Yes
IAG AU	Insurance Australia Group Ltd	Mar-14–Dec-19	Yes	Yes
TCL AU	Transurban Group	Mar-16–Dec-19	Yes	Yes
SCG AU	Scentre Group	Jun-14–Dec-19	No	No
FGL AU (1812574D AU)	Foster's Group Limited	Jan-10–Nov-11	No	No
WFD AU	Westfield Corporation	Jan-10–Apr-18	No	No
WRT AU	Westfield Retail Trust	Dec-10–Feb-11	No	No
S32 AU	South32 Ltd	May-15, Mar-18–Nov-19	No	No
1624320D AU	Unibail-Rodamco-Westfield	May-18	No	No
OMN AU	OneMarket Limited	May-18	No	No
URW AU	Unibail-Rodamco-Westfield CDI	May-18	No	No
1723503D AU	Amcor Ltd	Jun-18–May-19	No	No
COL AU	Coles Group Ltd	Nov-18–May-19	No	No
AMC AU	Amcor Plc	Jun-19–Dec-19	Yes	No
GMG AU	Goodman Group	Jun-19–Dec-19	Yes	No

**Fig. 3:**
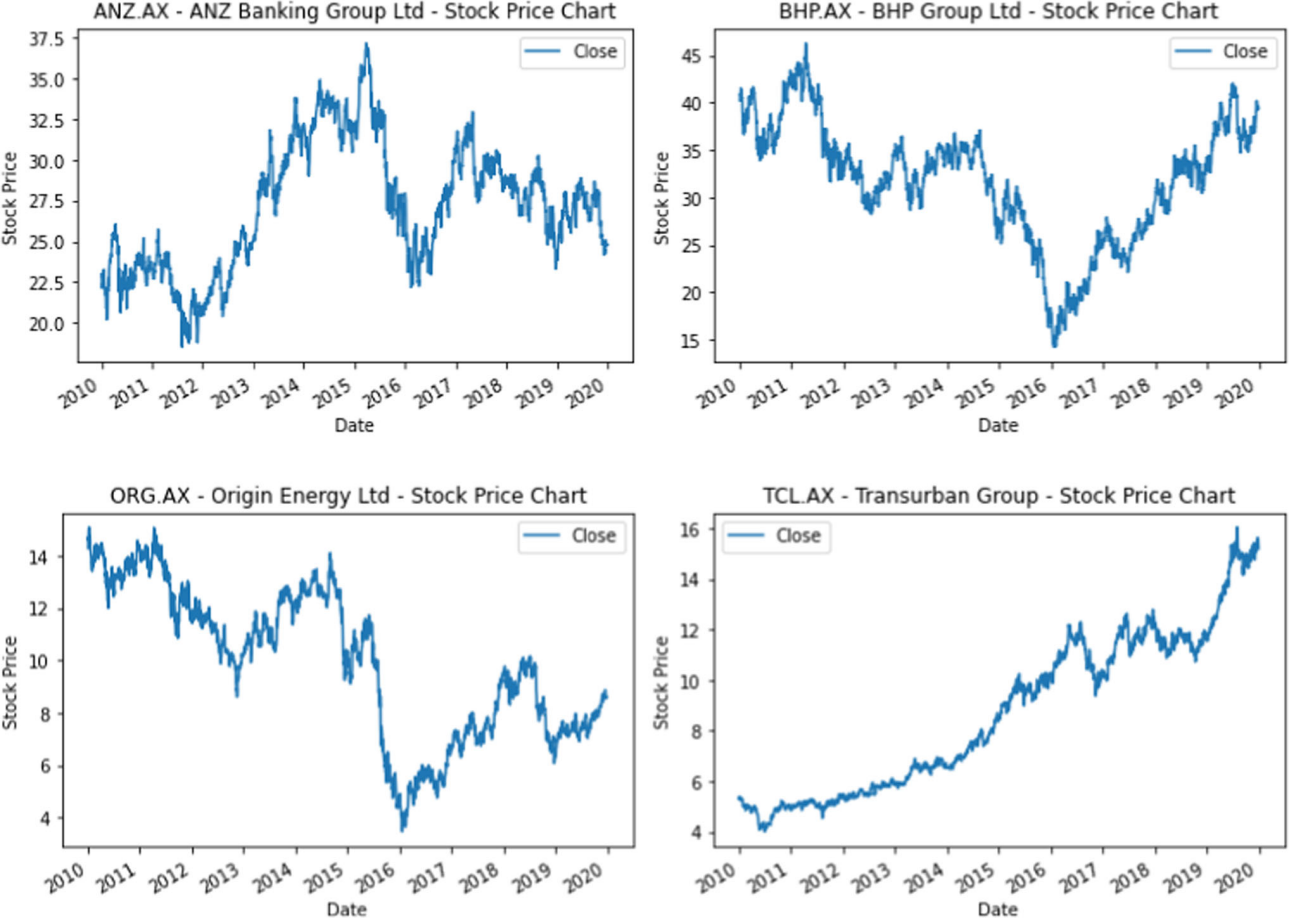
Example price charts of the stocks under analysis

For illustration purposes, example price charts for several stocks included in this study are provided below (Fig. 3).

This study diverges from the previous research (for example, Zhang [30] or Bisoi et al. [4, 5] by testing models on the stock price data that may have moved out of a certain narrow range (e.g., within 3 standard deviations of the historical mean). Dataset under study is chosen broadly to capture stock price movements of any kind and range to eliminate any bias from the research.

To present this data in a meaningful manner, we calculate technical indicators utilising the following parameters of a “lookback period” $L$ = 5 and an “forward period” $O$ = 1. These parameters have been chosen arbitrarily yet driven by the logic of having at least one week of daily stock price data (hence the lookback period of 5 days) and predicting for the time period ahead where this data sensibly matters (thus, limiting this to just 1 day ahead). The choice of parameters is consistent with the existing literature in the field (e.g., Göçken et al. [10], Khuwaja et al. [15]).

Using these parameters, the daily stock price data are then converted into a set of technical indicators as below. Assuming daily stock price data contains substantial amount of noise, Takens’ delay embedding theorem is relied upon to construct these smoothed “attractors” (i.e., technical indicators in this case) that could be used for stock price prediction Takens [26]. The technical indicators developed for this study were built to resemble the ones presented in the study by Zhang [30], yet with some addition, and comprise the following:

1. *Maximum*: the maximum stock price for the *i-*th lookback period as $Max({P}_{i-L}, {P}_{i-L+1},\dots , {P}_{i-1}, {P}_{i})$;

2. *Minimum*: the minimum stock price for the *i-*th lookback period as $Min({P}_{i-L}, {P}_{i-L+1},\dots , {P}_{i-1}, {P}_{i})$;

3. *Average*: the average value of the stock price for the *i-*th lookback period as $\frac{\sum \left({P}_{i-L}, {P}_{i-L+1},\dots , {P}_{i-1}, {P}_{i}\right)}{L}$;

4. *Standard Deviation (SD)*: the standard deviation of the stock price from its mean for the *i*-th lookback period calculated as $\sqrt{\frac{1}{L-1}\sum_{j=0}^{L}({P}_{i-j}-\overline{{P }_{i}})}$ where $\overline{{P }_{i}}$ is the mean of the stock price for the *i*-th lookback period (calculated as the *Average* above);

5. *Pseudo Log Return (PLR)*: the logarithmic difference between average prices of consecutive lookback periods;

6. *Trend Indicator (TI)*: a simple trend indicator calculated as the difference between the last close price for the *i*-th lookback period, ${P}_{i}$, and the first close price of the (*i*)-th lookback period, ${P}_{i-4}$, and taken as an “upward trend” (i.e., assigned the value of “1”) when the difference is positive (> 0), “no trend” (“0”) when there is no difference (i.e., ${P}_{i}$ = ${P}_{i-4}$), or “downward trend” when it is negative (< 0; and assigned the value of “-1”). This indicator is created to measure a trend within each lookback period;

7. *Previous Day Close (PDC)*: the stock price on the last day of the *i*-th lookback period.

Table 3 shows a sample set of technical indicators calculated for a stock given the original candlestick chart price data. As shown in Fig. 1, these technical indicators for each *i*-th lookback period (i.e., for each day in a sequence of daily stock price data) are fed into the ELM-trained SFNN model directly.

**Table 3:** Excerpt of the technical indicators calculated for a stock before standardisation

Date	Open	High	Low	Close	Adj Close	Volume	Max	Min	Average	StDev	PLR	Trend	TI	LastClose
2010–01-04	5.285	5.304	5.285	5.295	3.119	827,768							0	5.295
2010–01-05	5.343	5.400	5.285	5.400	3.181	2,228,606							0	5.400
2010–01-06	5.314	5.333	5.266	5.295	3.119	2,634,817							0	5.295
2010–01-07	5.304	5.314	5.285	5.314	3.131	2,313,408							0	5.314
2010–01-08	5.314	5.314	5.295	5.314	3.131	2,597,856	5.400	5.295	5.323	0.044		0.004	1	5.314
2010–01-11	5.295	5.314	5.285	5.304	3.125	1,356,795	5.400	5.295	5.325	0.042	0.000	− 0.018	− 1	5.304
2010–01-12	5.295	5.304	5.266	5.275	3.108	4,291,116	5.314	5.275	5.300	0.016	− 0.005	− 0.004	− 1	5.275
2010–01-13	5.266	5.295	5.266	5.266	3.102	3,662,073	5.314	5.266	5.295	0.022	− 0.001	− 0.009	− 1	5.266
2010–01-14	5.285	5.314	5.285	5.295	3.119	1,987,540	5.314	5.266	5.291	0.020	− 0.001	− 0.004	− 1	5.295
2010–01-15	5.304	5.304	5.285	5.304	3.125	2,712,298	5.304	5.266	5.289	0.017	0.000	0.000	0	5.304
2010–01-18	5.285	5.352	5.285	5.304	3.125	2,992,801	5.304	5.266	5.289	0.017	0.000	0.005	1	5.304
2010–01-19	5.314	5.333	5.295	5.295	3.119	4,173,939	5.304	5.266	5.293	0.016	0.001	0.005	1	5.295
2010–01-20	5.314	5.333	5.304	5.314	3.131	2,474,495	5.314	5.295	5.302	0.008	0.002	0.004	1	5.314
2010–01-21	5.333	5.343	5.304	5.323	3.136	5,513,970	5.323	5.295	5.308	0.011	0.001	0.004	1	5.323
2010–01-22	5.256	5.304	5.237	5.295	3.119	3,590,043	5.323	5.295	5.306	0.012	0.000	− 0.002	− 1	5.295
2010–01-25	5.266	5.266	5.189	5.237	3.086	2,221,040	5.323	5.237	5.293	0.033	− 0.003	− 0.011	− 1	5.237
2010–01-27	5.218	5.266	5.218	5.237	3.086	4,587,129	5.323	5.237	5.281	0.042	− 0.002	− 0.014	− 1	5.237
2010–01-28	5.218	5.256	5.065	5.132	3.023	5,624,977	5.323	5.132	5.245	0.073	− 0.007	− 0.036	− 1	5.132
2010–01-29	5.007	5.074	4.940	5.007	2.950	15,391,635	5.295	5.007	5.182	0.114	− 0.012	− 0.054	− 1	5.007
2010–02-01	5.017	5.055	4.960	5.027	2.961	7,658,040	5.237	5.007	5.128	0.110	− 0.010	− 0.040	− 1	5.027
2010–02-02	5.046	5.094	5.046	5.074	2.990	4,294,680	5.237	5.007	5.095	0.093	− 0.006	− 0.031	− 1	5.074
2010–02-03	5.046	5.084	5.027	5.027	2.961	2,923,179	5.132	5.007	5.053	0.050	− 0.008	− 0.021	− 1	5.027
2010–02-04	5.036	5.084	5.027	5.027	2.961	2,210,844	5.074	5.007	5.032	0.025	− 0.004	0.004	1	5.027
2010–02-05	5.007	5.055	4.950	4.998	2.944	2,331,890	5.074	4.998	5.030	0.028	0.000	− 0.006	− 1	4.998
2010–02-08	5.036	5.046	4.979	4.979	2.933	2,041,860	5.074	4.979	5.021	0.036	− 0.002	− 0.019	− 1	4.979
2010–02-09	4.931	5.017	4.931	4.988	2.939	4,343,396	5.027	4.979	5.004	0.022	− 0.003	− 0.008	− 1	4.988
2010–02-10	4.998	5.046	4.931	4.979	2.933	3,082,175	5.027	4.979	4.994	0.020	− 0.002	− 0.010	− 1	4.979
2010–02-11	4.988	5.074	4.960	5.027	2.961	2,242,066	5.027	4.979	4.994	0.020	0.000	0.006	1	5.027
2010–02-12	4.950	4.988	4.940	4.979	2.933	2,157,747	5.027	4.979	4.990	0.021	− 0.001	0.000	0	4.979
2010–02-15	4.969	4.979	4.912	4.960	2.922	1,013,593	5.027	4.960	4.986	0.025	− 0.001	− 0.006	− 1	4.960
2010–02-16	4.912	4.921	4.806	4.845	2.854	2,803,879	5.027	4.845	4.958	0.068	− 0.006	− 0.027	− 1	4.845
2010–02-17	4.835	4.902	4.691	4.883	2.877	4,802,653	5.027	4.845	4.938	0.074	− 0.004	− 0.029	− 1	4.883
2010–02-18	4.931	5.094	4.893	5.046	2.973	6,941,247	5.046	4.845	4.942	0.080	0.001	0.013	1	5.046
2010–02-19	5.065	5.141	4.998	5.046	2.973	4,504,954	5.046	4.845	4.956	0.092	0.003	0.017	1	5.046
2010–02-22	5.074	5.103	5.036	5.094	3.001	2,971,435	5.094	4.845	4.983	0.111	0.005	0.051	1	5.094

## Results and discussion

**Fig. 4:**
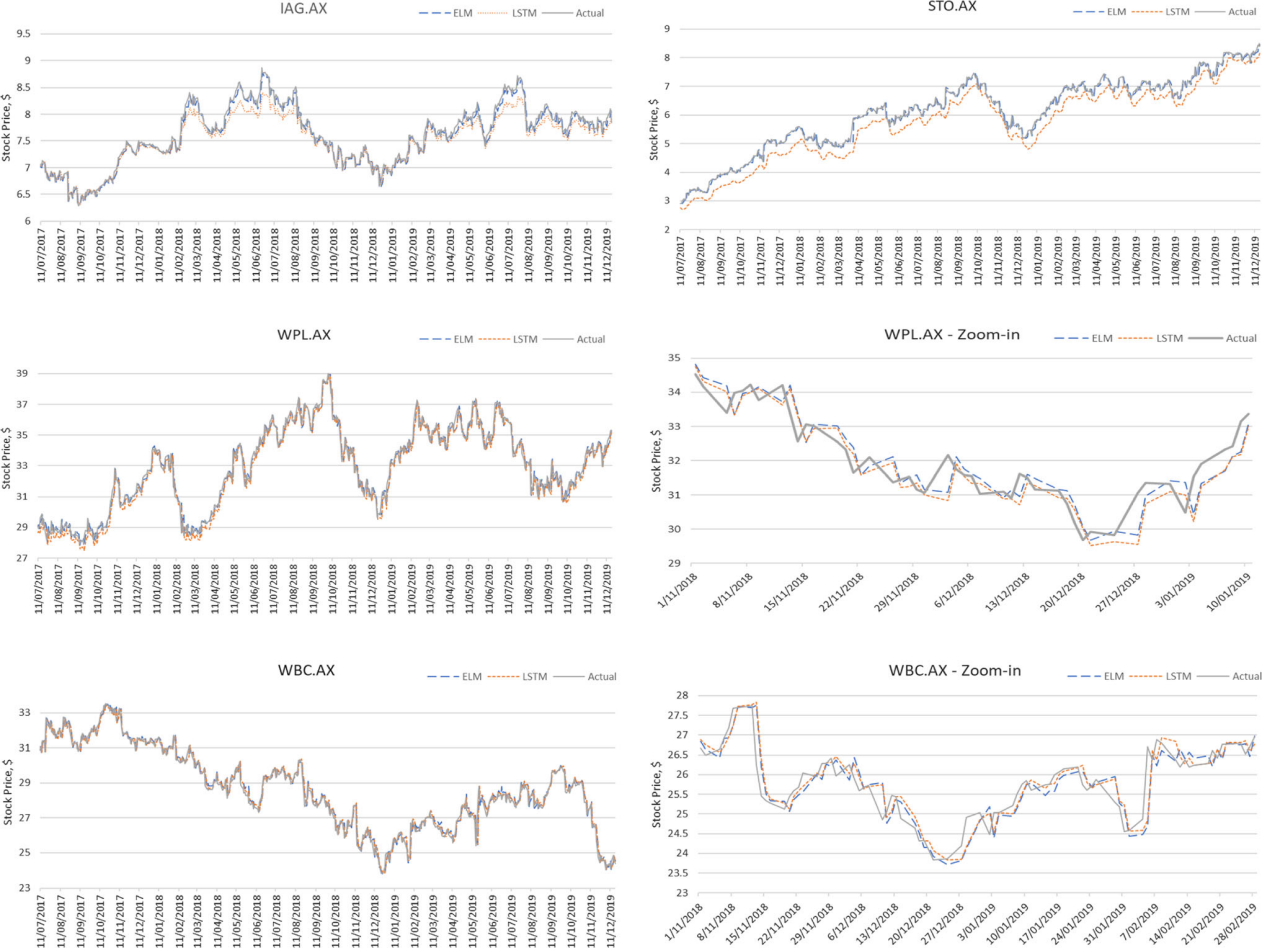
Time-series charts of prediction results of the models tested in comparison with the actual stock price data

Figure 4 visualises prediction results for a sample of stocks comparing two commonly used neural network training methodologies—ELM and LSTM—to the actual stock price data. This result is shown on the final, holdout test dataset (period from mid-2017 until the end of 2019) that has never been seen by any of the models and represents a one-off final test run. The original stock price (*grey*) is compared to the ELM-trained network prediction (*blue*) and prediction by the LSTM model (*orange*).

Summary numerical results for all stocks under analysis are presented in Table 4 on a number of critical performance metrics, including speed of training which gains importance as the prediction horizon decreases. All training and testing procedures were run on the Dell Latitude 5310 laptop with IntelI CITM) Processor, i5-10310U CPU @ 1.70 GHz, 2208 MHz, 4 Core(s), 8 Logical Processor(s) with 16.0 Gb of RAM (Installed Physical Memory).

**Table 4:** Comparative results for the models tested (lower error/better result **bolded**)

*#*		MSE		RMSE		Normalised RMSE		MAE		MAPE	
		LSTM	ELM	LSTM	ELM	LSTM	ELM	LSTM	ELM	LSTM	ELM
		(sec)	(sec)								
	Time taken to train and test the models	6232	56								
	*Ticker*										
*Group 1—ELM beats LSTM*											
1	AMP.AX	0.365	**0.020**	0.604	**0.142**	0.180	**0.042**	0.449	**0.104**	21.146	**4.387**
2	BHP.AX	0.280	**0.207**	0.529	**0.455**	0.016	**0.014**	0.420	**0.350**	1.275	**1.056**
3	BXB.AX	0.017	**0.016**	0.130	**0.127**	0.012	**0.012**	0.093	**0.090**	0.875	**0.837**
4	CBA.AX	0.660	**0.612**	0.813	**0.782**	0.011	**0.010**	0.627	**0.586**	0.828	**0.779**
5	CSL.AX	453.229	**7.088**	21.289	**2.662**	0.113	**0.014**	13.538	**1.925**	5.980	**1.011**
6	MQG.AX	60.481	**1.936**	7.777	**1.391**	0.068	**0.012**	6.288	**1.019**	5.059	**0.885**
7	ORG.AX	0.031	**0.017**	0.175	**0.129**	0.022	**0.016**	0.129	**0.098**	1.631	**1.215**
8	QBE.AX	0.030	**0.020**	0.174	**0.141**	0.015	**0.013**	0.136	**0.102**	1.247	**0.908**
9	RIO.AX	3.189	**1.860**	1.786	**1.364**	0.022	**0.017**	1.281	**1.077**	1.465	**1.303**
10	SUN.AX	0.024	**0.023**	0.154	**0.151**	0.011	**0.011**	0.109	**0.108**	0.781	**0.770**
11	TLS.AX	0.003	**0.003**	0.052	**0.051**	0.015	**0.015**	0.038	**0.035**	1.133	**1.042**
12	WES.AX	3.419	**0.401**	1.849	**0.633**	0.054	**0.019**	1.208	**0.469**	3.201	**1.306**
13	WOW.AX	0.095	**0.089**	0.309	**0.299**	0.010	**0.010**	0.232	**0.212**	0.770	**0.689**
14	STO.AX	0.053	**0.016**	0.230	**0.125**	0.038	**0.021**	0.185	**0.090**	3.173	**1.513**
15	IAG.AX	0.047	**0.018**	0.216	**0.135**	0.028	**0.018**	0.180	**0.106**	2.281	**1.358**
16	TCL.AX	0.170	**0.017**	0.412	**0.130**	0.033	**0.010**	0.304	**0.098**	2.232	**0.775**
AVERAGE		32.631	**0.771**	2.281	**0.545**	0.040	**0.016**	1.576	**0.404**	3.317	**1.240**
*Group 2 – LSTM beats ELM*											
17	ANZ.AX	**0.096**	0.099	**0.310**	0.315	**0.011**	0.011	**0.226**	0.232	**0.824**	0.846
18	NAB.AX	**0.077**	0.083	**0.278**	0.287	**0.010**	0.010	**0.200**	0.211	**0.729**	0.771
19	NCM.AX	**0.182**	0.194	**0.427**	0.440	**0.017**	0.018	**0.307**	0.322	**1.206**	1.284
20	WBC.AX	**0.111**	0.136	**0.333**	0.369	**0.012**	0.013	**0.236**	0.257	**0.835**	0.911
21	WPL.AX	**0.200**	0.233	**0.447**	0.483	**0.014**	0.015	**0.336**	0.365	**1.025**	1.102
AVERAGE		**0.133**	0.149	**0.359**	0.379	**0.013**	0.013	**0.261**	0.277	**0.924**	1.047

Overall, the performance of the ELM-trained models is exceeding that of the LSTM networks, with only a few cases of LSTM slightly outperforming the former. On average, Group 1 in Table 4—where ELM models provide superior prediction results to the LSTM ones—shows that ELM performs materially better than LSTM for these time-series. However, for Group 2—where LSTM yielded better performance than ELM—the difference in performance between the two models under study is minimal. This is also supported by the charts in Fig. 4 where the top two stocks (STO.AX and IAG.AX) belong to Group 1 and the other two stocks (WPL.AX and WBC.AX) are from Group 2. Group 1 stocks have visible difference in LSTM and ELM prediction, with LSTM being off point visibly. Group 2 sees ELM and LSTM predictions move almost too close to call.

The speed of training is also noteworthy to mention. ELM-trained models have been known to increase the speed of training by a factor of 10–100 (at least) compared to their more widely accepted counterparts (LSTM in this case) Wu et al. [28]. Our test showed ELM models have been trained and tested more than 100 times faster than LSTM ones for the full set of stocks under analysis. The value of this advantage is appreciated when dealing with constant flow of live data on shorter frequencies and the need to make prompt decisions (e.g., in the high-frequency trading environment).

In general, ELM appears to capture well trends in the data, as well as better reacting to more short-term changes in the price move process (i.e., peaks and troughs). This may be attributed to the set of technical indicators used for prediction where network is almost able to build an expected stock price distribution and choose the most likely next point given the latest point in the data (i.e., previous day close price).

Let us contrast the stocks where LSTM achieved better prediction results than ELM with the ones where it did not. They do belong to either the financial services (ANZ, NAB, WBC) or metals and mining (NCM, WPL) industries, however, stocks belonging to the same industries are found within Group 1 as well (for example, CBA, MQG or BHP, STO) where ELM performed better than LSTM. Therefore, a hypothesis that changes between these two groups may be driven by industry membership does not stand.

Table 5 presents initial further analysis on the issue. Descriptive statistics are calculated for stocks from the two groups, split by the dataset used to train, validate, and test the models. It should be noted that the final model is trained on the full combined training and validation dataset, before running the final test on the holdout dataset. This timewise split allows us to explore the differences in model performance between stocks.

**Table 5:** Descriptive statistics of the stock price time-series for the datasets used

#	Ticker	Training dataset				Validation dataset				Test dataset			
		Mean	Standard Deviation	Skewness	Kurtosis	Mean	Standard Deviation	Skewness	Kurtosis	Mean	Standard Deviation	Skewness	Kurtosis
*Group 1—ELM beats LSTM*													
1	AMP.AX	4.950	0.634	0.174	− 0.570	5.615	0.535	0.353	− 0.592	3.382	1.335	0.281	− 1.544
2	BHP.AX	35.026	3.896	0.525	− 0.262	23.198	4.172	− 0.167	− 0.871	33.093	4.415	− 0.085	− 0.884
3	BXB.AX	7.213	1.422	0.527	− 1.013	11.201	1.054	0.150	− 0.842	10.642	1.199	0.444	− 1.068
4	CBA.AX	60.923	12.017	0.478	− 1.353	80.171	5.945	0.467	− 0.565	75.789	4.449	− 0.045	− 1.069
5	CSL.AX	48.410	16.850	0.457	− 1.312	103.794	13.292	0.845	0.045	187.888	39.324	0.307	− 0.449
6	MQG.AX	39.933	11.964	0.265	− 1.174	78.193	8.141	− 0.598	− 0.215	114.701	14.778	− 0.456	− 0.919
7	ORG.AX	12.271	1.316	− 0.265	− 0.734	6.988	2.188	0.723	− 0.734	8.078	0.967	0.475	− 0.940
8	QBE.AX	14.727	3.152	0.767	− 0.093	12.129	1.363	− 0.177	− 0.965	11.212	1.017	0.070	− 1.203
9	RIO.AX	66.720	9.495	0.593	− 0.528	53.134	7.179	− 0.038	− 0.890	82.464	11.225	0.315	− 0.919
10	SUN.AX	10.593	2.371	0.512	− 1.320	13.392	0.883	− 0.208	− 0.581	14.066	0.679	0.643	0.350
11	TLS.AX	3.986	1.000	0.263	− 1.469	5.462	0.603	− 0.016	− 0.694	3.390	0.375	0.106	− 0.473
12	WES.AX	26.069	3.817	0.239	− 1.512	29.816	1.281	− 0.118	− 0.596	33.923	3.746	0.459	− 0.882
13	WOW.AX	29.795	4.108	0.393	− 1.338	25.294	2.817	0.678	0.072	30.083	3.881	0.878	− 0.329
14	STO.AX	11.434	1.195	− 0.885	1.671	4.681	1.312	0.822	− 0.648	6.077	1.297	− 0.540	− 0.383
15	IAG.AX	4.598	1.183	0.263	− 1.537	5.871	0.386	− 0.131	− 0.392	7.620	0.561	− 0.202	− 0.645
16	TCL.AX	5.879	1.009	0.579	− 0.468	10.443	0.971	0.287	− 0.913		1.427	0.870	− 0.779
*Group 2—LSTM beats ELM*													
17	ANZ.AX	26.058	4.471	***0.419***	− 1.254	28.830	3.641	***0.303***	− 0.712	27.662	1.536	*** − 0.364***	− 0.454
18	NAB.AX	26.587	4.119	***0.483***	− 1.354	29.957	3.232	***0.422***	− 0.596	27.719	2.231	*** − 0.027***	− 0.804
19	NCM.AX	25.064	11.550	*** − 0.146***	− 1.519	17.486	4.522	***0.120***	− 1.462	24.536	5.033	***1.177***	0.182
20	WBC.AX	26.436	4.990	***0.322***	− 1.428	32.155	2.530	***1.103***	0.793	28.696	2.281	***0.070***	− 0.773
21	WPL.AX	38.318	4.207	***0.113***	− 0.926	30.242	3.151	***0.174***	− 1.122	32.944	2.667	*** − 0.116***	− 0.970

First, it is worth noting that either model, but especially LSTM, performed comparatively worse on stocks that experienced strong growth through the period of analysis (2010–2019). For example, CSL.AX and MQG.AX had substantial increase in the mean between datasets. Second, it appears that stocks from Group 2 had substantial change in data distribution skewness (highlighted by bold italics in the table) between periods. For example, ANZ.AX showed positive skewness around 0.3–0.4 in the training and validation datasets which then retraced to a negative 0.36 in the test period. WPL.AX time-series followed the similar path to ANZ.AX. NAB.AX and WBC.AX showed matching trajectory where a positive skewness in the full training period changed to almost 0 in the test data. NCM.AX skewness has sharply increased from the negative 0.15 for training to the positive above 1 for the test datasets. This change in skewness represents material change in data interaction dynamics between time-series mean and standard deviation that were used as critical input variables into the ELM-trained models. This may explain the difference in predictive performance results between models for these stocks, however, bodes well for further research.

To test economic significance and practicality of the aforementioned findings, a simple trading strategy is developed using ELM model-based stock price predictions. Table 6 shows profitability results by threshold level δ that is used in the following manner:

$$a_{t}^{i} = \left\{ {\begin{array}{*{20}l} {BUY,~\Delta s_{t}^{i} \ge \delta } \hfill \\ {HOLD,~\delta > \Delta s_{t}^{i} \ge - \delta } \hfill \\ {SELL,~\Delta s_{t}^{i} < - \delta } \hfill \\ \end{array} ,} \right.$$

where $${a}_{t}^{i}$$ is the action taken at time $$t$$, $$\Delta {s}_{t}^{i}$$ is the expected change in stocks price $$s$$ of stock $$i$$ for time period $$t$$ based on the ELM model prediction, and δ is the chosen threshold level in absolute $ terms to indicate whether the predicted stock price change is considered material. Performance of the prediction-based strategy is tested on several threshold levels as there does not appear to be agreement in the literature on a common value or even an approach. For example, Mohanty et al. [19] and Bisoi et al. [4, 5] only compare the next day predicted price to the current day value, inherently assigning 0 value to the action threshold. We use threshold in this strategy to avoid unnecessary trades based on minor changes in price and, since no agreement appears to exist in the literature as to its value, we test a range of threshold levels.

**Table 6:** Profitability of the trading strategy based on the ELM model predictions, by threshold level δ

#	Ticker	*Threshold level, δ*											
		$0.01		$0.05		$0.10		$0.25		$0.50		$1	
		$	%	$	%	$	%	$	%	$	%	$	%
1	AMP.AX	0.44	− 56.15%	0.49	− 51.46%	0.62	− 37.58%	1.00	0.00%	1.00	0.00%	1.00	0.00%
2	ANZ.AX	0.81	− 19.28%	0.86	− 13.97%	0.76	− 24.37%	0.94	− 6.13%	1.00	0.00%	1.00	0.00%
3	BHP.AX	1.24	**24.26%**	1.17	**17.49%**	1.01	**0.67%**	1.15	**14.88%**	1.00	0.00%	1.00	0.00%
4	BXB.AX	1.17	**16.90%**	1.26	**25.90%**	1.00	0.00%	1.00	0.00%	1.00	0.00%	1.00	0.00%
5	CBA.AX	1.15	**15.31%**	1.14	**13.67%**	1.09	**8.98%**	1.15	**15.18%**	1.00	− 0.01%	1.00	0.00%
6	CSL.AX	1.74	**74.33%**	1.96	**96.31%**	1.92	**92.46%**	2.03	**103.07%**	1.79	**79.28%**	1.34	**34.26%**
7	MQG.AX	1.10	**10.13%**	1.09	**9.44%**	1.14	**14.03%**	1.24	**23.83%**	1.09	**9.47%**	1.16	**15.74%**
8	NAB.AX	1.02	**2.43%**	0.98	− 2.47%	1.06	**5.80%**	0.91	− 9.35%	1.00	0.00%	1.00	0.00%
9	NCM.AX	1.46	**45.62%**	1.38	**37.93%**	1.53	**53.26%**	1.32	**32.07%**	1.58	**57.88%**	1.19	**19.18%**
10	ORG.AX	1.36	**35.99%**	1.00	0.00%	1.00	0.00%	1.00	0.00%	1.00	0.00%	1.00	0.00%
11	QBE.AX	1.04	**4.06%**	1.09	**8.79%**	1.22	**22.21%**	1.00	0.00%	1.00	0.00%	1.00	0.00%
12	RIO.AX	1.01	**0.83%**	0.97	− 2.53%	1.02	**1.66%**	1.03	**3.49%**	1.19	**19.05%**	0.96	− 4.06%
13	SUN.AX	1.05	**5.44%**	1.00	0.00%	1.00	0.00%	1.00	0.00%	1.00	0.00%	1.00	0.00%
14	TLS.AX	0.87	− 13.27%	0.90	− 10.00%	1.00	0.00%	1.00	0.00%	1.00	0.00%	1.00	0.00%
15	WBC.AX	0.71	− 29.23%	0.62	− 38.28%	0.67	− 32.56%	0.80	− 20.02%	0.86	− 13.72%	1.00	0.00%
16	WES.AX	1.27	**27.40%**	1.19	**19.49%**	1.25	**24.83%**	1.01	**1.40%**	1.00	0.00%	1.00	0.00%
17	WOW.AX	1.35	**35.16%**	1.36	**35.83%**	1.45	**44.55%**	1.31	**31.43%**	1.00	0.00%	1.00	0.00%
18	WPL.AX	1.15	**15.04%**	1.16	**16.50%**	1.30	**29.81%**	1.29	**28.98%**	1.00	0.00%	1.00	0.00%
19	STO.AX	1.00	− 0.21%	1.44	**43.76%**	1.00	0.00%	1.00	0.00%	1.00	0.00%	1.00	0.00%
20	IAG.AX	1.04	**4.46%**	1.00	0.00%	1.00	0.00%	1.00	0.00%	1.00	0.00%	1.00	0.00%
21	TCL.AX	1.32	**31.83%**	1.35	34.68%	1.02	**2.44%**	1.00	0.00%	1.00	0.00%	1.00	0.00%
AVERAGE		1.11	**11.00%**	1.11	**11.48%**	1.10	**9.82%**	1.10	**10.42%**	1.07	**7.24%**	1.03	**3.10%**

This table reports profitability results of the trading strategy based on the ELM-trained neural network model predictions. The results are presented by threshold level used to identify action among BUY, HOLD, and SELL options. A range of threshold values has been used given lack of disagreement thereto in the literature. Profitable results are **bolded**. Average results at the bottom of the table are calculated as the overall profitability of a trading strategy that allocates the same dollar amount to trading each individual stock from the start, i.e., equal-weighted portfolio with no rebalancing

We test model performance using simple return metric by various thresholds to indicate persistence of the findings and conduct robustness checks. The main result of ELM outperformance over its LSTM counterpart holds across the full range of thresholds used. The difference is that fewer trades are made with higher thresholds. Profitable results are **bolded** in Table 6. In addition to being profitable for the majority of stocks, it is important to note that the overall average results of the model are positive across all threshold values. These results may be best understood as investing the same amount of funds for each individual stock trading from the beginning. These results are designed to reveal that real-world implementations are possible, and future research should investigate an optimal trading strategy based on such a model.

## Conclusion and future research

Prediction of future stock prices returns is arguably one of the most challenging areas of finance. This is driven by common belief that stock prices have a relatively low signal-to-noise ratio and a substantial array of influential factors, with time- and cross-section-varying weightings assigned to each. This presents a difficult task for predictive models, albeit an almost impossible one for traditional, linear models. Nonlinear machine learning models have shown some success in this field, but they most often require vast amounts of data to extract usable features from the high-noise price data. However, the relatively new Extreme Learning Machine training methodology for single hidden layer neural networks has not yet been comprehensively evaluated for this purpose. This study compares this relatively novel ELM training methodology to the more well-known Long Short-Term Memory (LSTM) recurrent neural network. Utilising a set of simple technical indicators built on raw stock price data, ELM-trained neural networks are constructed, trained, and then tested on a set of blue-chip stocks listed on the Australian Securities Exchange.

The analysis confirms the proposed benefits of ELM training, specifically reduced training time with comparable predictive power without requiring as much data. It further confirms emerging findings of Zhang [29] as to the overall efficacy of the models and potential shortfalls, albeit on a broader real stock price dataset. ELM-trained models have shown substantial improvement in predictive accuracy on the majority of individual stock price datasets used in the study. In the relatively few cases where LSTM does better, it appears that changes in the stock price data distribution might be the reason for this deviation.

The performance discrepancy between LSTM and ELM-trained models bodes well for potential future research. There are relatively few number of cases where LSTM outperforms and, in these cases, we have observed substantial drift in the degree of skewness between training and test datasets as compared to less unexpected changes for the rest of the stock price series. LSTM’s ability to capture changes in the underlying asymmetry of the distribution may prove a significant advantage in practice. Identifying important variables in the ELM neural networks and trialling additional input metrics directly relating to skewness of the input series distribution also represent interesting research directions. Additionally, testing the results on a different set of financial instruments may shed further light on the findings about the LSTM’s ability to respond better to changes in the underlying data distribution which would be a useful feature in investment practice.

Given the encouraging results, future research could investigate ways to further enhance the ELM-based trading system. It would be valuable to investigate adding a stronger trend indicator (such as a medium or a long-term Exponential Moving Average) given that the current ELM did not perform well with the changing distribution of the price data. It is likely to help better identify the underlying trend in the data and adjust price prediction accordingly. Another parameter that could be further tuned is the threshold value in the action decision step. The strategy presented used an initial set of dollar-based thresholds, but the optimal threshold to use might vary according to underlying timeseries data and its distribution properties. Finally, the choice of input variables used in the model can be further investigated to improve its predictive power. For example, it might be beneficial to trial various volatility measures instead of the standard deviation currently used in the model. Measures that may better reflect more recent volatility or more accurately capture both short- and long-term inherent price series variance are most promising. Such future research identifying better methods to more accurately predict security prices has widespread applications, from improving on pure profitability-driven investment objectives to better informed portfolio construction and risk management.

## Data Availability

The datasets generated during and/or analysed during the current study are available in the Yahoo Finance repository, https://finance.yahoo.com/lookup?s=DATA. Data have been sourced using the API module connection direct from Python IDE, Spyder using the yahoo-finance module commands. Additionally, the datasets generated during and/or analysed during the current study are also available from the corresponding author on reasonable request.

## Footnotes

1 Suganthan and Katuwal [24] provide an overview of original works in the area of randomisation-based feedforward neural networks, shedding light on potential origins of the ELM training methodology.
